# Persistent Gaps in Disability-Free Life Expectancy Among Older Adults with Mental Illness

**DOI:** 10.1093/geroni/igaf122.3638

**Published:** 2025-12-31

**Authors:** Yuelin Li, Yuelong Xu, Zhirui Deng, Rezvaneh Manzour, Kyeongra Yang, Yue Coco Dong, Heeyoung Lee

**Affiliations:** University of PIttsburgh, Pittsburgh, Macau; University of Pittsburgh, Pittsburgh, Pennsylvania, United States; University of Pittsburgh, Pittsburgh, Pennsylvania, United States; University of Pittsburgh, Pittsburgh, Pennsylvania, United States; Rutgers University, Newark, New Jersey, United States; University of Pittsburgh, Pittsburgh, Pennsylvania, United States; University of Pittsburgh, Pittsburgh, Pennsylvania, United States

## Abstract

Mental illness affects about 14% of U.S. adults aged 50 and older and may compromise healthy aging, yet its long-term impact on disability-free life expectancy (DFLE) remains underexamined. This study estimated and compared DFLE by mental illness status, assessed temporal trends, and evaluated transitions between healthy and disabled states. Using nationally representative data from the U.S. Health and Retirement Study (1994–2020; N = 14,553), we applied multi-state Markov models to estimate DFLE for adults without mental illness (Neither), with either depressive symptoms or a psychiatric history (Either), or with both (Both). In models adjusted for age and sex, DFLE at baseline was 32.37 years for the Neither group, 26.75 years for the Either group, and 21.76 years for the Both group; by 2020, it declined to 19.87, 15.53, and 13.77 years, respectively. Annual decline rates were similar across groups (Neither = –1.13; Either = –1.02; Both = –0.92 years per wave). Data showed that individuals with mental illness transitioned from a healthy state to disability (transition intensity 0.15 to 0.12/person-year) at approximately twice the rate of those in the general population (transition intensity: 0.06/person-year), while disability-to-health transition rates were similar across groups. The findings indicate the Either and Both groups experience earlier aging-related decline, yet functional recovery is possible even with mental illness. Overall, mental illness was consistently associated with shorter DFLE and longer disabled years, highlighting the need for integrated care for people with mental illness that effectively address both mental and physical health across the care settings.

